# Community-Associated Methicillin-Resistant *Staphylococcus aureus* Clonal Complex 80 Type IV (CC80-MRSA-IV) Isolated from the Middle East: A Heterogeneous Expanding Clonal Lineage

**DOI:** 10.1371/journal.pone.0103715

**Published:** 2014-07-31

**Authors:** Houda H. Harastani, Sima T. Tokajian

**Affiliations:** Department of Natural Sciences, School of Arts and Sciences, Lebanese American University, Byblos, Lebanon; Rockefeller University, United States of America

## Abstract

**Background:**

The emergence of community-associated methicillin resistant *Staphylococcus aureus* (CA-MRSA) has caused a change in MRSA epidemiology worldwide. In the Middle East, the persistent spread of CA-MRSA isolates that were associated with multilocus sequence type (MLST) clonal complex 80 and with staphylococcal cassette chromosome *mec* (SCC*mec*) type IV (CC80-MRSA-IV), calls for novel approaches for infection control that would limit its spread.

**Methodology/Principal Findings:**

In this study, the epidemiology of CC80-MRSA-IV was investigated in Jordan and Lebanon retrospectively covering the period from 2000 to 2011. Ninety-four *S. aureus* isolates, 63 (67%) collected from Lebanon and 31 (33%) collected from Jordan were included in this study. More than half of the isolates (56%) were associated with skin and soft tissue infections (SSTIs), and 73 (78%) were Panton-Valentine Leukocidin (PVL) positive. Majority of the isolates (84%) carried the gene for exofoliative toxin d (*etd*), 19% had the Toxic Shock Syndrome Toxin-1 gene (*tst*), and seven isolates from Jordan had a rare combination being positive for both *tst* and PVL genes. *spa* typing showed the prevalence of type t044 (85%) and pulsed-field gel electrophoresis (PFGE) recognized 21 different patterns. Antimicrobial susceptibility testing showed the prevalence (36%) of a unique resistant profile, which included resistance to streptomycin, kanamycin, and fusidic acid (SKF profile).

**Conclusions:**

The genetic diversity among the CC80 isolates observed in this study poses an additional challenge to infection control of CA-MRSA epidemics. CA-MRSA related to ST80 in the Middle East was distinguished in this study from the ones described in other countries. Genetic diversity observed, which may be due to mutations and differences in the antibiotic regimens between countries may have led to the development of heterogeneous strains. Hence, it is difficult to maintain “the European CA-MRSA clone” as a uniform clone and it is better to designate as CC80-MRSA-IV isolates.

## Introduction

*Staphylococcus aureus*, a highly adaptive and versatile gram-positive bacterium, is considered one of the most isolated human pathogens and the most common cause of skin and soft tissue infections (SSTIs) [1], [2]. Soon after the introduction of methicillin for the treatment of penicillin-resistant strains in 1959, methicillin-resistant *S. aureus* (MRSA) has emerged as an important hospital-associated (HA-MRSA) pathogen for its increased morbidity and mortality rates, healthcare costs, and length of hospital stay [3], [4]. HA-MRSA infections arise in individuals with predisposing risk factors, such as surgery or presence of an indwelling medical device. By contrast, many community-associated MRSA (CA-MRSA) infections arise in otherwise healthy individuals who do not have such risk factors. CA-MRSA infections are also known to be epidemic in some countries. These features suggest that CA-MRSA strains are more virulent and transmissible than are traditional HA-MRSA strains [5].

CA-MRSA lineages are genotypically and phenotypically unrelated to the former multi-drug resistant HA-MRSA, and recently have started replacing the once pandemic HA-MRSA clones (CC5, 8, 22, 36, and 45) in health care facilities [6], [7]. Continent-specific-PVL positive CA-MRSA clones were previously described: ST1-IV (USA400), ST8-IV (USA300), ST30-IV (Pacific/Oceania), ST59-IV/V (USA1000, Taiwan), and ST80-IV (European CA-MRSA) [8], which were also reported from other parts of the world [9], [10], [11], [12], [13].

Common to all lineages is that they are generally susceptible to non-β-lactam antibiotics, harbor the small-sized staphylococcal chromosomal cassette *mec* (SCC*mec*) types IV or V encoding methicillin resistance, and carry the Panton-Valentine Leukocidin (PVL) toxin genes [2], [14], [15]. European clone ST80-IV (allelic profile 1-3-1-14-11-51-10) was first identified in the early 1990s and today is found throughout Europe, the Middle East, and Northern Africa [10], [16], [17], [18], [19], [20]. This clonal lineage is PVL-positive, belongs to *agr* type III, has type 8 capsular polysaccharide, and is resistant to tetracycline, streptomycin, kanamycin, and fusidic acid with a pronounced susceptibility to gentamicin [18], [19], [20].

Compared to the CA-MRSA clone most common in the United States (USA300), the European CA-MRSA clone seems less well adapted to persist in hospital environments, where CC80-MRSA-IV has entered Danish hospitals but has not caused nosocomial infections [18], [21]. Several reports indicated the transmission of the ST80-IV clone to Europe from patients with a recent history of travel or family relation to the Mediterranean or Middle East [18], [22], [23], [24], [25].

In the Middle East, little information is available about the emergence and continuous spread of CC80 clone. Khalil *et al.* [12] performed molecular characterization of 103 *S. aureus* isolates (41 MRSA and 62 MSSA) recovered from stool and nose specimens collected from children admitted to the University of Jordan Hospital. Genotyping revealed 48 different *spa* types and identified distinct allelic profiles with the majority belonging to ST80. On the other hand molecular characterization of 130 *S. aureus* clinical isolates (93 MRSA and 37 MSSA) recovered from patients at the Clinical Microbiology Section of the American University of Beirut in Lebanon revealed the presence of 48 *spa* types that clustered into 30 different groups. MLST revealed 10 sequence types (STs) among the isolates, and the majority of the PVL-positive isolates (53%) were ST80-MRSA-IVc [10]. However, a similar more recent study was conducted on 132 *S. aureus* non-duplicate clinical isolates recovered in a period of six months at AUB-MC [11]. MRSA represented 30% of the isolates collected in this study, with the most common being: t021 (6%), t044 (5%), and t267 (5%). Clustering SCC*mec* with MLST identified seven MRSA and 20 MSSA clones, and confirmed that the PVL-positive ST80-MRSA-IV was the dominant clone in Lebanon. The present retrospective study provides data on the epidemiology and molecular characteristics of ninety-four CC80-MRSA-IV isolates collected from Lebanon and Jordan over an 11-year period (2000–2011).

## Materials and Methods

### Ethical approval

Ethical approval was not required as clinical isolates were collected and stored as part of routine clinical care. Clinical isolates and patient records/information were anonymized and de-identified prior to analysis.

### Hospital setting

Isolates from Jordan were obtained from the University of Jordan Hospital (UJH) in Amman, a governmental hospital that serves over 500,000 patients annually with a 547-inpatient bed capacity, while those from Lebanon were collected from the American University of Beirut Medical Center (AUB-MC) in Beirut, a private university hospital that provides tertiary services for over 300,000 patients annually with a 350-inpatient bed capacity.

### Clinical Isolates

*S. aureus* isolates (*n* = 478) were collected from Lebanon and Jordan from 2000 to 2011. Isolates were confirmed as *S. aureus* by Gram staining, positive catalase reaction, and production of coagulase enzyme using SLIDEX Staph Plus agglutination kit (Biomérieux, France). All isolates identified to be s*pa* type t044 and/or belonging to *spa*-clonal cluster 044 (*spa-*CC 044), were included in this study. In total 94 isolates (Jordan, *n = *31; Lebanon, *n = *63) were undertaken in this study. DNA was extracted using a Nucleospin Tissue kit (Macherey-Nagel, Germany) according to manufacturer’s instructions.

### Clinical and demographic information

Clinical and demographic data were extracted from patients’ charts and lab discharge summaries and included: specimen origin (skin and soft tissue, respiratory, blood, stool, etc.), age, gender, time of isolation and hospitalization criteria (in or out-patient, surgery, etc.).

### Antibiotic susceptibility testing

Antimicrobial susceptibility testing was performed using the Kirby-Baüer disk diffusion method according to Clinical and Laboratory Standards Institute (CLSI) recommendations [28] for streptomycin, kanamycin, tetracycline, gentamicin, fusidic acid, penicillin G, rifampicin, erythromycin, and clindamycin. Discs were purchased from Oxoid (Oxoids, UK) and Biorad (Bio-Rad, Marnes-la-Coquette, France) and samples were streaked on Muller-Hinton agar plates (Oxoids, UK) with an 18–20 hour incubation at 35±1°C. Resistance for fusidic acid (< 24 mm) was determined according to breakpoints defined by the European Committee on Antimicrobial Susceptibility Testing (EUCAST) v3.0. *S. aureus* ATCC 29213 was used as a quality control strain to determine assay sensitivity.

### Multiplex PCR (M-PCR) for detection of 16S rRNA, PVL, and *mec*A genes

Amplification of the 16S rRNA that served as an internal positive control, PVL (*lukS*-PV and *lukF*-PV), and *mec*A genes were done as described by McClure *et al.* [29]. PVL negative MRSA (N315) and PVL positive MSSA (ATCC 49775) were used as controls. The amplification reaction contained 1.5 µl of template DNA in a final volume of 25 µl containing 0.4, 0.8 and 0.8 µM for the primers specific for the 16S rRNA, *lukS-PV,* and *mecA* genes respectively with 2U of AmpliTaq (Fermentas), 1.5 mmol/l MgCl2, 1.6x Taq buffer, 0.2mM of each deoxynucleotide triphosphate (dNTP). The thermocycling conditions were set at 94°C for 5 min followed by 10 cycles of 94°C for 45 s, 55°C for 45 s, and 72°C for 75 s and 25 cycles of 94°C for 45 s, 50°C for 45 s, and 72°C for 75 s PCR products were resolved in a 1.8% (w/v) Metaphor (Lonza, Rockland, ME, USA) agarose gel in 0.5% Tris-borate-EDTA buffer (Bio-Rad, Hercules, CA) at 80 V/cm for 1 hour and were visualized with ethidium bromide.

### Toxin gene profiling

Presence of exofoliative toxins a (*eta*), b (*etb*), and d (*etd*), and staphylococcal Toxic Shock Syndrome Toxin 1 (*tst*) genes was determined using previously described PCR primers [29], [30], [31] using a single M-PCR reaction. A Qiagen multiplex PCR kit was used where conditions were first optimized using the following reference strains: TC-142 (*eta* positive), TC-7 (*etb* positive), and NCTC11963 (*tst* positive). Reaction mixtures contained 1 µg of chromosomal template, 25 µl master mix with 3 mM MgCl_2_, 5 µl primer mix (2 mM in TE buffer for each primer) and RNase-free water to a final volume of 50 µl. The optimal cycling conditions were as follows: 95°C for 15 min; 30 cycles of 94°C for 30 s, 57°C for 1.5 min, and 72°C for 1.5 min; and a final extension at 72°C for 10 min.

### MRSA SCC*mec* typing

SCC*mec* elements were typed using previously described PCR primers [32]. For multiplex PCR, a Qiagen multiplex PCR kit was used, and conditions were optimized using the following reference strains: MRSA NCTC 10442 (SCC*mec* I), MRSA N315 (SCC*mec* II), MRSA 85/2082 (SCC*mec* III), MRSA JCSC 4744 (SCC*mec* IVa), MRSA JCSC 2172 (SCC*mec* IVb), MRSA JCSC 47882 (SCC*mec* IVc), and MRSA WIS (SCC*mec* V) as previously described [32], [33]. Reaction mixtures contained 1 µg of chromosomal template, 25 µl master mix with 3 mM MgCl_2_, 5 µl primer mix (2 mM in TE buffer for each primer) and RNase-free water to a final volume of 50 µl. The optimal cycling conditions were as follows: 95°C for 15 min; 30 cycles of 94°C for 30 s, 57°C for 1.5 min, and 72°C for 1.5 min; and a final extension at 72°C for 10 min.

### *spa* typing

The polymorphic X region of *Staphylococcus* protein A (spa) was amplified for all isolates as previously described [34], [35].

### Multilocus sequence typing (MLST)

Twenty-four representative isolates were typed by MLST to confirm their relatedness to the CC80 clone. The isolates were selected based on variation of specimen origin, year of isolation and covering all different *spa* types within *spa*-CC 044. Amplification of the seven housekeeping genes (*arcC*, *aroE*, *glpF*, *gmk*, *pta*, *tpi*, and *yqiL*) by MLST was done as previously described [36].

### PFGE fingerprinting

All isolates were subjected to PFGE typing using *Sma*I as previously described [37]. A bacteriophage lambda ladder PFG marker (New England BioLabs, UK) was included in each gel and NCTC 8325 was used as a quality control reference strain.

### Data Analysis

*spa* types were assigned using Ridom Staph Type v2.2.1 database (Ridom GmbH, Würzburg, Germany) (www.ridom.de/spaserver/) and clustered into *spa* clonal clusters (*spa*-CCs) using the algorithm based upon repeat pattern (BURP) with clustering parameters excluding *spa* types with fewer than five repeats and grouping *spa* types to the same *spa*-CC if the cost was ≤4. CLC main workbench software v6.8.4 (CLC bio, Denmark) was used to assemble and align sequences of the seven housekeeping genes and sequence types (STs) were determined by submitting the allelic profile of representative alleles to the MLST database (http://saureus.mlst.net/) and eBURST v3.0 software was used to determine the clonal relationship of the isolates with the entire MLST database. PFGE fingerprints obtained were compared by means of Dice coefficient, and cluster analysis was performed by the unweighted pair group method with arithmetic means (UPGMA) using GelCompar and Bionumerics software v6.5 (Applied Maths, Sint-Martens-Latem, Belgium) with 1% band tolerance and 0.5% optimization settings. Groups were clustered according to the recommendations of Tenover *et al.* [26] and by applying a similarity coefficient of 80% to all dendrograms as suggested by Struelens *et al.* [27].

Categorical comparisons were performed using Chi-square test (X^2^). A *P* value of less than 0.05 was considered to be significant. The associations between resistance patterns with the sample origin, gender and site of infection were evaluated using the R statistical package (v. 3.0.2). The associations of the presence and absence of the toxins: *eta, etb, etd*, TSST-1 and PVL with the sample origin, gender and site of infection were also evaluated. The function used in R include “chisq.test()” from the package “stats”.

## Results

### Study Population

Ninety-four MRSA isolates identified to be s*pa* type t044 and/or belonging to *spa*-clonal cluster 044 (*spa-*CC 044), and possibly related to ST80 were included in this study. Isolates were recovered from Lebanon (*n = *63/94 isolates; 67%) and Jordan (*n = *31/94; 33%) from 2000 to 2011 (Table 1 and Table 2). Around 56% of the isolates were associated with SSTIs, 15% with respiratory tract infections and 9% with bacteremia. Thirty-one of the isolates were from Jordan and 63 from Lebanon. Overall, 39% (*n* = 37/94) of the isolates were from females and 61% (*n* = 57/94) from males.

**Table 1:** Demographics and molecular characteristics of isolates collected from Lebanon.

Site ofinfection	Gender	Age^1^	*spa*Type	MLST^2^	ToxinProfiling^3^	AntibioticProfile^4^
Wound	F	1	t044	80	*etd*, PVL	STR, KAN, TET, FUS, ERY, DA
Wound	M	47	t044	80	*eta*, *etd*, PVL	STR, KAN
Pus	F	11	t044	80	*etd*, PVL	STR, KAN, TET, FUS
Wound	M	1M	t044	80	*etd*, PVL	STR, KAN, FUS
Eye	F	70	t131	80	PVL	STR, KAN, FUS
Biopsy	M	71	t4222	80	*etd*, PVL	STR, KAN, TET, FUS
Wound	F	18	t044	80	*etd*, PVL	STR, KAN, FUS, ERY, DA
DTA	M	64	t044	80	*etd*	TET, FUS
Pus	M	9	t6438	80	*etd*, PVL	STR, KAN, FUS
Bronchial Lavage	M	47	t044	80	*etd*, PVL	STR, KAN, FUS
Others	F	24	t044	80	*etd*, PVL	STR, KAN, TET, FUS
Abscess	M	19	t131	80	*etd*, PVL	Sensitive to all tested antibiotics
Tracheal Aspirate	M	6M	t021	80	*etd*	STR, KAN, FUS
Wound	F	36	t9135	80	*etd*, PVL	STR, KAN, FUS
Abscess	M	41	t044	ND	*etd*, PVL	STR, KAN, FUS
Abscess	F	34	t044	ND	*etd*, PVL	STR, KAN, FUS
DTA	M	50	t044	ND	*etd*, PVL	STR, KAN, FUS
Abscess	M	31	t044	ND	PVL	STR, KAN, FUS
Abscess	F	29	t044	ND	*etd*, PVL	STR, KAN, FUS
Abscess	F	62	t044	ND	*etd*, PVL	STR, KAN, FUS
Wound	M	18	t044	ND	*etd*, PVL	FUS
DTA	M	60	t044	ND	*etd*, PVL	Sensitive to all tested antibiotics
Wound	M	4	t044	ND	*etd*, PVL	STR, KAN, TET, FUS
Wound	F	57	t044	ND	*etd*, PVL	STR, KAN, FUS
Wound	M	35	t044	ND	*etd*, PVL	STR, KAN, TET, FUS, ERY
Wound	F	52	t044	ND	*etd*, PVL	STR, KAN, TET, FUS, ERY
Wound	M	35	t044	ND	*etd*, PVL	STR, KAN, TET, FUS
Wound	M	21	t044	ND	*etd*, PVL	STR, KAN, TET, FUS
Wound	M	74	t044	ND	*etd*	STR, KAN, FUS
Wound	F	27	t131	ND	*etd*, PVL	STR, KAN, FUS
Blood	M	72	t044	ND	*etd*, PVL	STR, KAN, TET, FUS
Wound	M	53	t044	ND	PVL	STR, KAN, FUS
Wound	M	30	t044	ND	*etd*	STR, KAN, TET, GEN, FUS, ERY, DA
Catheter	F	89	t044	ND	*etd*, PVL	STR, KAN, FUS
Wound	F	41	t044	ND	*etd*, PVL	STR, KAN, FUS, ERY
Wound	M	82	t044	ND	PVL	STR, KAN, FUS
DTA	M	72	t044	ND	*etd*, PVL	STR, KAN, FUS, ERY
Wound	M	78	t044	ND	*etd*, PVL	STR, KAN, TET, FUS, ERY
Wound	F	42	t044	ND	*etd*, PVL	STR, KAN, TET, FUS
Wound	F	38	t044	ND	*etd*, PVL	STR, KAN, TET, FUS
Wound	F	63	t044	ND	PVL	STR, KAN, FUS
DTA	M	74	t044	ND	*etd*, PVL	STR, KAN, FUS
Wound	M	21	t044	ND	*etd*, PVL	STR, KAN, TET, FUS, ERY
Pus	F	27	t044	ND	*etd*, PVL	STR, KAN, FUS, ERY
Wound	M	1	t044	ND	*etd*, PVL	STR, KAN, FUS
Pus	M	46	t044	ND	*etd*, PVL	STR, KAN, FUS, ERY
Wound	F	24	t044	ND	*etd*, PVL	STR, KAN, FUS, ERY
Blood	M	60	t044	ND	*etd*, PVL	STR, KAN, FUS
Cyst	M	20	t044	ND	*etd*, PVL	FUS
Wound	M	58	t044	ND	*etd*, PVL	FUS
Wound	F	68	t044	ND	*etd*, PVL	STR, KAN, FUS
Pus	F	59	t044	ND	*etd*, PVL	STR, KAN, TET, FUS
Abscess	F	20	t044	ND	*etd*, PVL	STR, KAN, FUS, ERY
Biopsy	M	72	t044	ND	PVL	STR, KAN, FUS
Pus	M	47	t044	ND	*etd*	STR, KAN, FUS
Ear	F	45	t044	ND	*etd*, PVL	TET, FUS, ERY
Abscess	M	28	t044	ND	PVL	STR, KAN, TET, FUS
Pus	F	89	t044	ND	PVL	STR, KAN, FUS
Eye	F	4W	t131	ND	PVL	TET, FUS
Eye	M	4W	t131	ND	PVL	TET, FUS
Abdominal Fluid	M	24	t044	ND	*etd*, PVL	STR, KAN
Pus	F	18	t044	ND	*etd*, PVL	STR, KAN, TET, FUS
Abscess	M	24	t044	ND	*etd*, PVL	STR, KAN

1 W: weeks; M: months.

2 ND: non-determinant.

3 *eta*: exofoliative toxin a gene; *etd*: exofoliative toxin d gene; PVL: Panton-Valentine Leukocidin gene.

4 STR: streptomycin; KAN: kanamycin; TET: tetracycline; GEN: gentamicin; FUS: fusidic acid; ERY: erythromycin; DA: clindamycin.

**Table 2:** Demographics and molecular characteristics of isolates collected from Jordan.

Site ofinfection	Gender	Age^1^	*Spa*Type	MLST^2^	ToxinProfile^3^	AntibioticProfile^4^
**Nose**	F	4M	t044	80	*etd*, *tst*	STR, KAN, FUS, ERY
**Stool**	M	12M	t044	80	*etd*, *tst*	STR, KAN, FUS
**Nose**	M	16D	t044	80	*etd, tst*, PVL	STR, KAN, TET, FUS, ERY
**Nose**	M	12M	t131	80	*etd*, *tst*	STR, KAN, FUS
**Nose**	M	1M	t5849	80	*etd*, *tst*	STR, KAN, FUS, ERY
**Wound**	F	31	t044	80	*etd*, PVL	STR, KAN, TET, FUS
**Wound**	M	22	t044	80	*etd, tst*, PVL	STR, KAN, FUS, ERY
**Ear**	M	17	t5802	80	PVL	STR, KAN, FUS, ERY
**Pus**	F	12	t5849	80	*etd*, PVL	STR, KAN, FUS, ERY
**Gall Bladder**	M	25	t044	997	*etd, tst*, PVL	STR, KAN, TET, FUS, ERY, DA
**Stool**	M	26D	t044	ND	-	STR, KAN, FUS, ERY
**Stool**	F	4M	t044	ND	*etd*, *tst*	STR, KAN, FUS, ERY
**Nose**	M	12M	t044	ND	*etd*, *tst*	STR, KAN, FUS, ERY
**Stool**	M	1M	t044	ND	*etd*, *tst*	STR, KAN, FUS
**Stool**	M	2M	t044	ND	*etd*, *tst*	STR, KAN, FUS, ERY
**Nose**	F	15D	t044	ND	*etd*, *tst*	STR, KAN, FUS
**Stool**	F	15D	t044	ND	*etd*, *tst*	STR, KAN, FUS
**Nose**	M	15D	t044	ND	*etd*, *tst*	STR, KAN
**Abdominal Fluid**	F	89	t044	ND	*etd, tst*, PVL	STR, KAN, TET, FUS
**Gall Bladder**	F	37	t044	ND	*etd*, PVL	STR, KAN, FUS, ERY
**Blood**	M	33	t044	ND	*etd*, PVL	STR, KAN, TET, FUS, ERY
**Blood**	M	29	t044	ND	*-*	TET, FUS
**Blood**	F	43	t044	ND	*etd*, PVL	STR, KAN, FUS
**Peritoneal Fluid**	M	45	t044	ND	*-*	ERY
**Blood**	M	66	t044	ND	*-*	FUS, ERY
**Pus**	M	1D	t044	ND	*etd, tst*, PVL	STR, KAN, FUS, ERY
**Wound**	F	35	t044	ND	*etd*, PVL	STR, KAN, FUS, ERY, DA
**Blood**	M	52	t044	ND	*etd, tst*, PVL	STR, KAN, FUS
**Nose**	M	40	t044	ND	*etd*, PVL	STR, KAN, FUS
**Wound**	F	35	t044	ND	*etd, tst*, PVL	STR, KAN, FUS, ERY, DA
**Blood**	M	64	t6438	ND	*etd*	ERY, DA

1 D: days; W: weeks; M: months.

2 ND: non-determinant.

3 *etd*: exofoliative toxin d gene; *tst*: toxic shock syndrome toxin 1 gene; PVL: Panton-Valentine Leukocidin gene.

4 STR: streptomycin; KAN: kanamycin; TET: tetracycline; FUS: fusidic acid; ERY: erythromycin; DA: clindamycin.

### Characteristics of the MRSA clones

**Figure 1:**
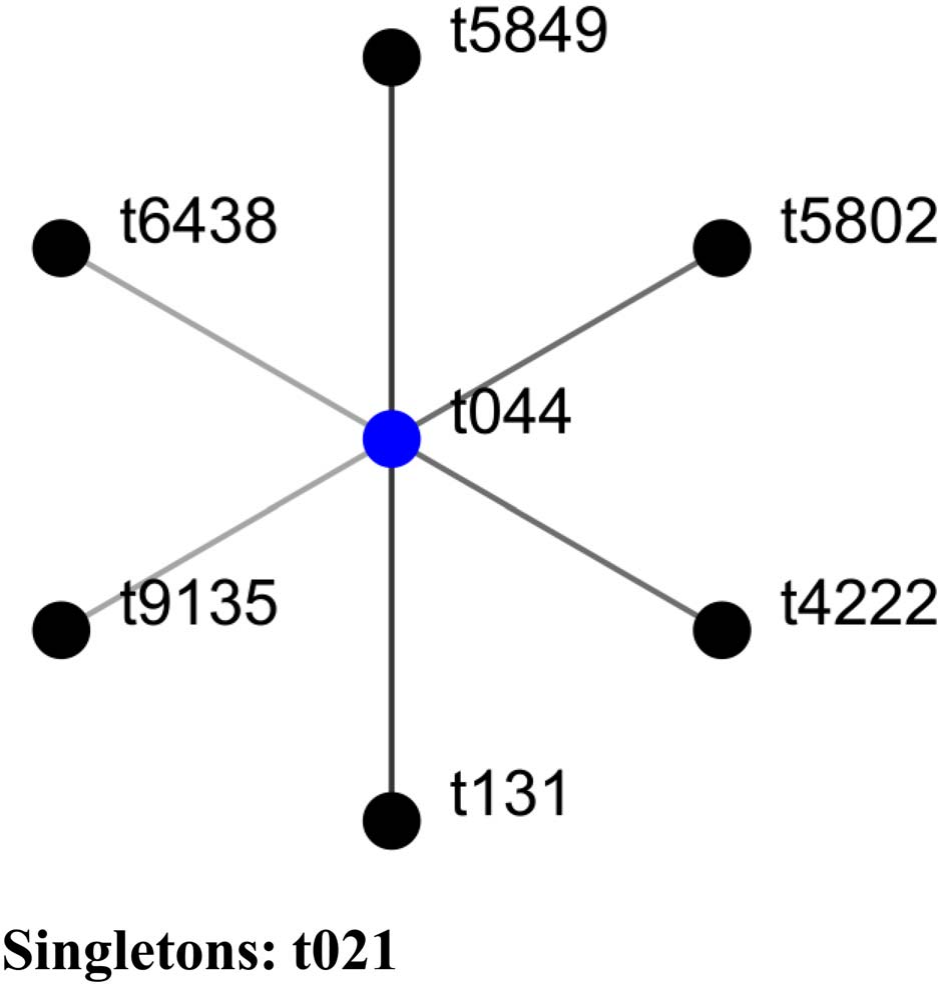
Population snapshot based on BURP analysis of all recovered *spa* types. Each dot represents a unique *spa* type. The diameter of the a dot is proportional to the quantity of the corresponding *spa* type. Blue dot represents group founder, defined as the *spa* type with the highest founder score within a CC.

*spa* typing of all 94 isolates revealed that the majority (85%) were of *spa* type t044 (Jordan *n* = 26/32; Lebanon *n* = 54/63) followed by single locus variants (SLV) of type t044 (t131, t5802, t5849, and t4222) or t131 (t5802), double locus variants (DLV) of type t044 (t6438 and t9135) and a singleton (t021) (Figure 1).

The isolates selected for MLST typing were chosen based on variation of specimen origin, year of isolation and covering all different *spa* types within *spa*-CC 044 (Table 1 and Table 2). MLST typing of these isolates showed that all belonged to ST80 except for one from Jordan, which was ST997. ST997 however, is also within the CC80 and is a SLV from ST80 (http://saureus.mlst.net/eburst/database.asp). This isolate, which was recovered in 2009 from Jordan, was positive for the PVL, *etd*, and *tst* genes and resistant to streptomycin, kanamycin, fusidic acid, erythromycin, and clindamycin (Table 2).

### Antibiotic Susceptibility

In this study the common resistance pattern observed was that of SKF (*n = *34/96; 36%) (Table 3). Resistance against streptomycin, kanamycin and fusidic acid was comparable regardless of the source (Jordan or Lebanon) being 86, 86 and 91%, respectively. A significant difference was detected between Jordan and Lebanon with respect to resistance to tertracycline (*p* = 0.0017), with the ones from Lebanon showing a higher resistance rate (*n* = 22/63; 35% Lebanon vs. *n* = 6/31; 19% Jordan). In contrast, the resistance rate against erythromycin within isolates recovered from Jordan (*n = *19/31; 61%) was higher compared to those from Lebanon (*n = *14/63; 22%) and the difference was also significant (*p* = 0.000565).

**Table 3:** Percentage distribution of resistance patterns.

Profilenumber	Antibiogram^1^	Lebanon (%)	Jordan (%)	Total (%)
1	STR, KAN, FUS	26 (41)	8 (26)	34 (36)
2	STR, KAN, FUS, ERY	6 (10)	11 (35)	17 (18)
3	STR, KAN, TET, FUS	12 (19)	2 (6)	14 (15)
4	STR, KAN, TET, FUS, ERY	4 (6)	2 (6)	6 (6)
5	TET, FUS	3 (5)	1 (3)	4 (4)
6	STR, KAN	3 (5)	1 (3)	4 (4)
7	STR, KAN, FUS, ERY, DA	1 (2)	2 (6)	3 (3)
8	FUS	3 (5)	-	3 (3)
9	STR, KAN, TET, FUS, ERY, DA	1 (2)	1 (3)	2 (2)
10	TET, FUS, ERY	1 (2)	-	1 (1)
11	STR, KAN, TET, GEN, FUS, ERY, DA	1 (2)	-	1 (1)
12	FUS, ERY	-	1 (3)	1 (1)
13	ERY, DA	-	1 (3)	1 (1)
14	ERY	-	1 (3)	1 (1)

1 STR: streptomycin; KAN: kanamycin; TET: tetracycline; GEN: gentamicin; FUS: fusidic acid; ERY: erythromycin; DA: clindamycin.

### Toxins

PVL genes were detected in 78% (*n = *73/94) of the isolates, with 52% (*n = *16/31) of the isolates from Jordan and 8% (*n = *5/63) from Lebanon being PVL-negative (Table 1 and Table 2). e*ta toxin* gene was only detected in one isolate recovered from wound in Lebanon, which was additionally PVL positive, while none was positive for *etb*. Genetic diversity was additionally observed between the two set of isolates (Jordan vs. Lebanon) with the *etd* and the *tst* genes, where 64% of the isolates from Lebanon were positive for *etd* gene and none for *tst* compared to 28% for *etd* gene and 19% for *tst* in isolates from Jordan. PVL and TSST-1 were both found to be significantly associated to the sample origin (PVL: *p* = 6.295e-06 and TSST-1: *p* = 1.136e-10), PVL mainly detected in isolates from Lebanon and TSST-1 only in isolates from Jordan, and with the site of infection (PVL: *p* = 8.726e-05 and TSST-1: *p* = 0.0009773); being higher in isolates from wound, pus, and abscess versus all other sites of infection. Both toxin genes were not significantly associated to gender and none of the remaining toxins was significantly associated to the origin, gender, or site of infection. Seven of the isolates, all collected from Jordan, were positive for PVL, *tst* and *etd* genes. Six of the isolates were recovered in 2008 and only one in 2009. There was no correlation between the isolates’ resistance and PFGE patterns.

One PVL-negative isolate from Lebanon had the common European antibiotic resistance pattern (TSKF) with additional resistance to gentamicin, clindamycin, and erythromycin. *tst* positive isolates on the other hand, in addition to being resistant to the β -lactam drugs were resistant to streptomycin, kanamycin, and fusidic acid. *spa* typing of all the seven *tst* positive and MLST typing of three revealed that all were *spa* type t044, two were ST80 and one ST977, with all belonging to the CC80 lineage. Finally, a clear heterogeneity was detected within the other studied toxin genes too, with 64% of the isolates from Lebanon being positive for *etd* gene compared to only 28% for those from Jordan (Table 1 and Table 2).

Overall, 16% (*n* = 15/94) of the isolates had the same genetic characteristics as that of the European ST80 (*etd* positive, PVL positive, SCC*mec*-IV and TSKF resistance pattern). It is noteworthy that all 15 isolates were recovered from Lebanon (Table 1).

### PFGE

**Figure 2:**
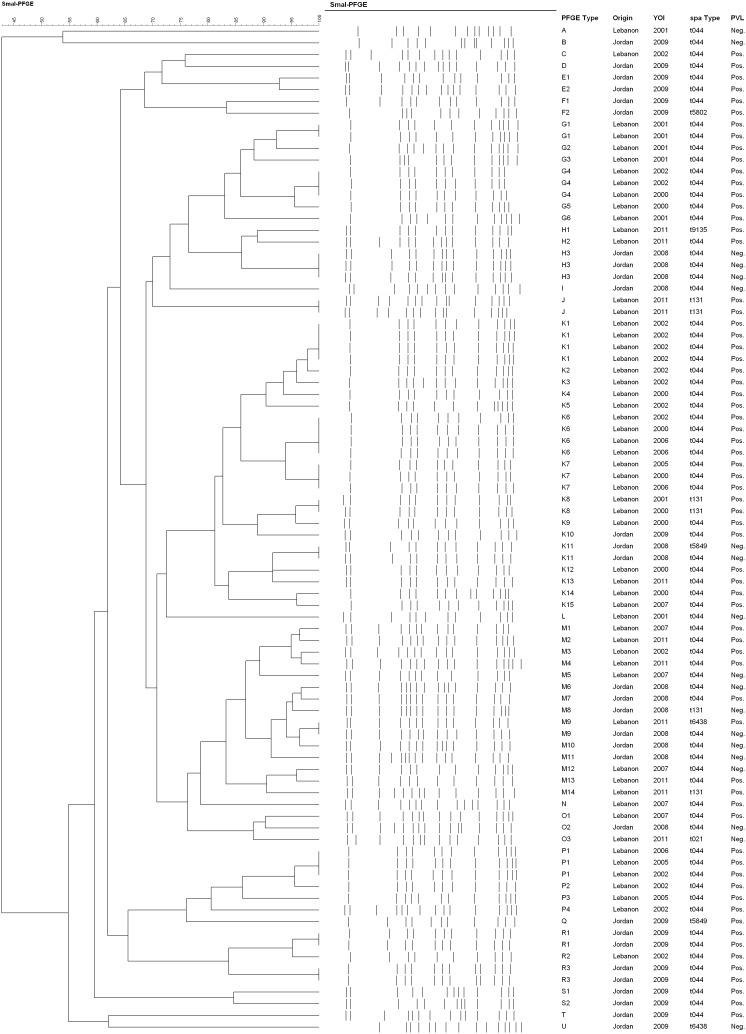
Dendrogram of PFGE clusters of CC80 isolates. *Sma*I macrorestriction patterns were analyzed using the Dice coefficient and visualized by un-weighted pair group method (UPGMA), using average linkages with 1% tolerance and 1% optimization settings. PFGE groups determined by cluster analysis are numbered from A-U. Origin, year of isolation (YOI), *spa* type, and PVL status of each isolate is also included.

PFGE-based analysis clustered the 94 isolates in 21 different clonal groups when employing 80% as a similarity cutoff value, with 26% of the isolates clustering in one group designated as clonal group K (Figure 2, Table 1 and Table 2). This pulsotype had isolates from both countries, and all except for two isolates from Jordan were PVL positive, and were of *spa* types: t044, t131, and t5849. The genetic diversity occurred during the whole study period with isolates from both countries showing diversification and at times coexistence. The diversity between the isolates recovered from both countries however, was again clearly seen with the lack of any common pulsotype. Finally, different *spa* types, resistance profiles, and toxin genes did not correlate with specific PFGE subtypes.

## Discussion

In Europe, most CA-MRSA isolates were associated with CC80-IV with the first report detecting an ST80-IV isolate being in 1998 in Greece [38]. Since then, sporadic ST80-IV cases have been reported in many European countries, which argued the possibility of the clone being introduced from the Middle East [18], [21], [25], [39]. Geographically, the Middle East is a heterogeneous region composed of 17 countries that vary substantially in terms of size, population and culture. Several reports from the Middle East have previously detected and reported the circulation of ST80-IV clone [10], [12], [40], [41], [42], [43], [44], [45]. Understanding the ST80-IV epidemic in the Middle East and its potential successful transmission to Europe was an important endeavor towards better control. Accordingly this study was conducted, which included a collection of projected CC80 related MRSA recovered from Lebanon (*n = *63/94 isolates; 67%) and Jordan (*n = *31/94; 33%) from 2000 to 2011, in an attempt to determine the relatedness, if it exists, between the European ST80-IV and the ones prevalent in the Middle East.

CA-MRSA have been associated primarily with community acquired infections, predominantly SSTIs, in young people [2]. Having 56% of the isolates in this study associated with SSTIs agrees with the notion of ST80-IV isolates being primarily associated with SSTIs in patients outside hospitals [46]. However, isolates causing invasive infections, including bacteremia (9%) and respiratory tract infections (15%) were also detected and included.

The most common antimicrobial resistance pattern observed within ST80 isolates circulating in Europe is the one against tetracycline, streptomycin, kanamycin and fusidic acid (TSKF pattern) [18], [47]. A different common pattern however, was detected among the isolates examined in this study, being mainly that of SKF (*n = *34/96; 36%). Resistance against streptomycin, kanamycin and fusidic acid was comparable regardless of the source (Jordan or Lebanon) being 86, 86 and 91%, respectively. Compared to the European ST80 isolates, we detected in general a higher susceptibility to tetracycline especially with the ones recovered from Jordan (Lebanon *n* = 22/63; 35% resistant vs. *n* = 6/31; 19% for Jordan). On the other hand, higher resistance to erythromycin was detected within the isolates from Jordan (*n = *19/31; 61%) as compared to those from Lebanon (*n = *14/63; 22%). Similarly, Udo and Srakhoo [45] also showed the presence of variations in the resistance patterns between isolates recovered from Kuwait when compared to the European ST80 clone. This diversity reflects differences in the treatment regimens that exist between those countries.

PVL, a prophage-encoded bi-component pore-forming protein, is encoded by two genes: *lukS-PV* and *lukF-PV* residing in genomes of some bacteriophages (e.g.: ΦSa2958, ΦSa2MW, ΦPVL) and these are readily transferrable following selective bacterial infection [48]. At elevated concentrations, PVL causes host cell lysis; however at lower concentrations, PVL primes neutrophils to release inflammatory mediators such as leukotriene B4, IL-8, granule contents and reactive oxygen species [49]. Although its role in pathogenicity remains controversial, many murine-conducted studies show the role of PVL in mitochondrial inactivation and apoptosis as well as its association with certain established diseases such as necrotizing pneumonia and SSTIs [2], [50], [51]. PVL genes were detected in 78% (*n = *73/94) of the isolates. Contrary to the European PVL-positive ST80, 52% (*n = *16/31) of the isolates from Jordan and 8% (*n = *5/63) from Lebanon were PVL-negative. PVL-negative ST80 was previously detected in Kuwait [45], Algeria [52], Switzerland and France [53]. Whether these isolates arose from PVL positive ones due to the loss of the PVL phage or represent native ST80 backgrounds that had not previously acquired the PVL phage is something that needs to be further clarified. It is however noteworthy, that a PVL-negative isolate from Lebanon had the common European antibiotic resistance pattern (TSKF) with additional resistance to gentamicin, clindamycin, and erythromycin. This finding was in line with the study conducted by Ramdani-Bouguessa *et al.* [54] from Algeria, thus posing a problem of having a possibility of ST80 invading hospital settings that adds an additional significant threat to public health. Finally, two out of the four isolates that were both PVL and *etd* negative were recovered from blood and were of *spa* type t044 and SCC*mec*-IV. Previously Adler et al. [40] demonstrated that PVL negative and SCC*mec*-IV *S. aureus* isolates were associated with pediatric HA-MRSA bloodstream infections.

Another significant finding adding up to the observed genetic diversity within the isolates undertaken in this study at one hand and the European ST80-IV on the other, was the detection of both *tst* and PVL genes in seven of the isolates that were recovered from Jordan. TSST-1 is a superantigen that stimulates the release of large amounts of proinflammatory factors in human infection, has been associated with human toxic shock syndrome [55], and causes sepsis by uncontrolled stimulation of T lymphocytes triggering a cytokine storm [56]. TSST-1 element is carried on a pathogenicity island known now as Sapl1, carrying the *tst* and other virulence factors [57]. A single strain of *S. aureus* rarely produces both PVL and TSST-1 [58]. However, Holmes *et al.* [59] previously documented the *tst* genes in four of 30 PVL-positive isolates. All the isolates were typed and belonged to lineages CC30, CC5 and CC22 some of which were multi-drug resistant. Similarly, *tst* positive isolates in this study, in addition to being resistant to the β-lactam drugs were resistant to streptomycin, kanamycin, and fusidic acid. *spa* typing of all the seven and MLST typing of three revealed that all were *spa* t044, two were ST80 and one ST997, with all belonging to the CC80 lineage. Our finding was in line with a recent study in Jordan in which two putative ST80-IV isolates belonging to *spa* type t044 harbored both the PVL and *tst* genes [41]. Zhi *et al.* [58] showed that a PVL-carrying phage from strain MSSA 68111, which was positive for both PVL and *tst* genes, was a variant of icosahedral-head type phage FPVL. These findings suggested that FPVL and FPVLv68111 might have evolved from a common ancestor and that genetic drift may have occurred in one or both. Features unique to FPVLv68111 may have permitted MSSA68111 to acquire the genes for TSST-1 production. Whether a similar genetic drift led to having isolates positive for both toxins in those isolates from Jordan needs to be further investigated, specially that it was a significant deviation from the norm and that it indicated the emergence of hypervirulent *S. aureus* strains. Finally, a clear heterogeneity was additionally detected within the other studied toxin genes, with 64% of the isolates from Lebanon being positive for *etd* gene compared to only 28% for those from Jordan; detecting *etd* toxin gene is a common finding within the European ST80 isolates [18], [53], [60], which emphasizes again that ST80-IV should be considered as a clonal lineage. Yamaguchi *et al.* [61], showed that the *etd* gene was carried on a pathogenicity island and hypothesized that ETD may play a pathogenic role in a variety of infections by destroying epithelial barriers, helping bacteria to spread or invade tissues. This could partly explain the success of the isolates within some of the ST80 isolates, which usually carried the gene for ETD in combination with the gene for PVL [59].

The most common *spa* type so far detected within the CC80-MRSA-IV isolates has been type t044 (*spa* repeat pattern r07 r23 r12 r34 r34 r33 r34) in Europe [53], [62], [63], [64], [65], Africa [17], [20], [43], [66], and Asia [10], [12], [44]. *spa* typing of the isolates in this study revealed the following types: t044, single locus variants (SLV) of type t044 (t131, t5802, t5849, and t4222) or t131 (t5802), double locus variants (DLV) of type t044 (t6438 and t9135) and a singleton (t021 annotated to CC30). *spa* type t131 was frequently reported among CC80-MRSA-IV isolates from Europe [18], [21], [64], [65]. It is noteworthy however, that one isolate within the *spa* type t131, which also belonged to the ST80-IV and was positive for PVL and *etd* genes, showed no resistance to any of the tested antibiotics. This was in contrast to what was recently reported by Hadjihannas *et al.* [65], with a similar isolate recovered from Greece belonging to the ST80-IVc and *spa* type (t131), but expressing an increase in resistance repertoire to include five different classes of non–β-lactam antibiotics, namely, quinolones, macrolides, clindamycin, fusidic acid, and tetracyclines again re-emphasizing the existing genetic diversity within ST80-IV clonal lineage.

All isolates chosen for MLST typing, based on variation of specimen origin, year of isolation and covering all different *spa* types within *spa*-CC 044, were ST80 except for one from Jordan, which was ST997. ST997 however, is also within the CC80 and is a SLV from ST80 (http://saureus.mlst.net/eburst/database.asp).

PFGE-based analysis clustered the 94 isolates in 21 different clonal groups when employing 80% as a similarity cutoff value, with 26% of the isolates clustering in one group designated as clonal group K. This pulsotype had isolates from both countries, all except for two isolates from Jordan were PVL positive, and isolates were of *spa* types: t044, t131, and t5849. Different *spa* types, resistance profiles, and toxin genes did not correlate with specific PFGE subtypes. The diversity observed within the isolates recovered from both countries along with the lack of any common pulsotype, diminishes the possibility of cross transmission.

European CA-MRSA has previously been described as a rather uniform clone**.** However, the high degree of molecular diversity observed in recent years, and being additionally supported by the diversity observed in this study, makes it difficult to maintain “the European CA-MRSA clone” as a uniform clone, and it is better to refer to them, and as suggested previously, as CC80-MRSA-IV isolates [18]. Close surveillance of these strains is essential to monitor their spread, antimicrobial resistance profiles, and association with disease. Finally, the successful expansion of ST80 and the heterogeneity observed in this study calls for novel approaches in infection control measures to monitor their spread.
